# Mortality impacts of long-term PM_2.5_ and NO_2_ exposure in Great Britain under national and international air quality limits

**DOI:** 10.1016/j.apr.2025.102827

**Published:** 2026-04

**Authors:** Gillian Flower, Rochelle Schneider, Karen Exley, Christina Mitsakou, Pierre Masselot, Antonio Gasparrini

**Affiliations:** a Environment & Health Modelling (EHM) Lab, Department of Public Health, Environments and Society, London School of Hygiene & Tropical Medicine, London, United Kingdom; b Department for Health and Social Care, UK Government, United Kingdom; c Φ-lab, European Space Agency (ESA), Frascati, Italy; d Epidemiology and Population Health, London School of Hygiene & Tropical Medicine, London, United Kingdom; e Air Quality and Public Health, Environmental Hazards and Emergencies Department, UK Health Security Agency, United Kingdom

**Keywords:** Air pollution, Mortality, Britain, WHO, PM_2.5_, NO_2_

## Abstract

PM_2.5_ and NO_2_ are two of the most dangerous air pollutants, contributing to substantial excess mortality across Europe. In 2021, the World Health Organization (WHO) revised its air quality (AQ) guidelines, decreasing the recommended annual limit from 10 μg/m^3^ to 5 μg/m^3^ for PM_2.5_, and from 40 μg/m^3^ to 10 μg/m^3^ for NO_2_.

In this study, we analysed concentrations and associated long-term premature mortality across Great Britain from 2008 to 2018 in relation to multiple AQ limits, as determined by WHO guidelines and international/national regulations.

The percentage of the population exposed to PM_2.5_ concentrations above 10 μg/m^3^ decreased from 67.6 % to 38.2 % over the study period, whereas those exposed to NO_2_ levels above 40 μg/m^3^ remained low. Levels below the new, lower WHO guidelines for PM_2.5_ and NO_2_ were reached in only a handful of locations.

Exposure to PM_2.5_ in 2018 resulted in 36,403 (30,422–42,640) premature deaths, whereas NO_2_ exposure contributed to 20,175 (7125–32,281) premature deaths. These numbers could be reduced by 46 % and 44 %, respectively, if exceedances beyond the lower cut-offs were avoided.

These findings indicate that complying with the new WHO recommendations would require considerable effort, but they can lead to substantial health benefits.

## 1 Introduction

Long-term exposure to outdoor air pollution is a leading environmental risk factor. (WHO (World Health Organization), 2024, EEA (European Environment Agency), 2023a, Strak et al., 2021; Chen and Hoek, 2020) Fine particulate matter with an aerodynamic diameter smaller than 2.5 μm (PM_2.5_) and nitrogen dioxide (NO_2_), among the most hazardous pollutants, contribute to substantial excess mortality in Europe with an estimated 253,000 and 52,000 deaths respectively, in 2021 alone. (EEA (European Environment Agency), 2023b, Liu et al., 2019; Nazarenko et al., 2021; Cohen et al., 2017; McDuffie et al., 2021) In the UK, it is estimated that long-term PM_2.5_ and NO_2_ exposure was responsible for between 26,287 and 42,442 deaths in 2018 (Macintyre et al., 2023).

International organisations and national governments have responded by enacting air quality guidelines and regulations that set maximum limits om the concentrations of various pollutants. In 2005, the World Health Organization (WHO) published the Global Air Quality Guidelines (AQGs), (WHO (World Health Organization), 2016b, WHO (World Health Organization), 2016a, WHO (World Health Organization), 2005) which recommended that annual average exposures do not exceed 10 μg/m^3^ of PM_2.5_ and 40 μg/m^3^ of NO_2_. In 2021, these thresholds were reduced to 5 μg/m^3^ and 10 μg/m^3^, respectively. (WHO (World Health Organization), 2024) Less strict levels have been imposed by the European Union’s (EU) Air Quality Directive (AQD) with an annual average PM_2.5_ limit of 25 μg/m^3^ from 2015 to 20 μg/m^3^ from 2020, (EU (European Union), 2008) whereas the annual average NO_2_ limit has remained at 40 μg/m^3^ since 2010. In the United Kingdom (UK), the Air Quality Standards (AQS) also set the annual average NO_2_ concentration limit as 40 μg/m^3^. In 2023, new legislation for fine particulate matter introduced two environmental targets for PM_2.5_ in England, to be achieved by 2040: (1) a maximum annual mean concentration target of 10 μg/m^3^, and (2) a population exposure reduction of 35 % compared to 2018. (UK Government, 2023, DEFRA (UK Department for Environment Food & Rural Affairs)) Prior to this, the UK AQS imposed a 20 μg/m^3^ limit to PM_2.5_ concentration to be met by 2020 in England, Wales, and Northern Ireland. (DEFRA (UK Department for Environment Food & Rural Affairs)) In Scotland, the UK AQS determined a limit of 10 μg/m^3^ to be met by 2020. (DEFRA (UK Department for Environment Food & Rural Affairs)) A summary of the air quality limits is provided in Supplementary Table S1.

The evaluation and potential revision of AQ targets must be informed by epidemiological evidence. Several studies have assessed the geographical and temporal variation in air pollution across the UK, as well as the associated excess mortality (UK Government, 2022b; UK Government, 2022a; UK Government, 2018; Khomenko et al., 2021; Milojevic et al., 2017; Carnell et al., 2019; Dajnak et al., 2021). Published investigations have reported associations between PM_2.5_ and NO_2_ related mortality at concentrations below the current limits (Strak et al., 2021; Khomenko et al., 2021; Di et al., 2017; Brauer et al., 2019; Qian et al., 2021), suggesting a health benefit of lower concentration limits. However, no study thus far has attempted to quantify the health benefits associated with the implementation of multiple existing or new concentration targets in the UK. This process is vital for providing a comprehensive assessment to inform policy decisions, balanced against other socioeconomic considerations (Jbaily et al., 2022). In addition, air pollutant concentrations show strong geographical and temporal differences, as well as variations between urban and rural areas, therefore the assessment of compliance with any new AQ limits should account for local differences in pollution concentrations.

This study addresses this gap, first by assessing population exposure to PM_2.5_ and NO_2_ from 2008 to 2018 via high spatial resolution coverage of Great Britain, and then by quantifying the related mortality impact under scenarios of compliance with a range of potential concentration limits.

## 2 Materials and methods

### 2.1 Small area data

Great Britain comprises the countries of England, Scotland, and Wales with a total population of more than 61 million in 2011 (Table 1). (ONS (UK Office for National Statistics), 2011a) This study used geographical units defined by small census-based areas to determine the annual pollutant exposure and associated excess mortality. The boundaries of these units were independently defined by countries within the UK to form administrative divisions for the 2011 census. (NISRA (Northern Ireland Statistics and Research Agency), 2015) In England and Wales, the units are 34,753 (32,844 and 1,909, respectively) lower layer super output areas (LSOAs) containing, on average, 1600 residents. In Scotland, they are represented by 6976 Data Zones (DZs), including, on average, 760 people. LSOAs and DZs were used to define small areas (SAs) and assign annual PM_2.5_ and NO_2_ exposure, and other area-level characteristics for the computation of excess mortality. Specifically, population estimates for each SA stratified by five-year time intervals of 13 age groups (from 0 to 4 to 90 years and older) were obtained from the 2011 census from NOMIS. (ONS (UK Office for National Statistics), 2011b).

For the computation of the baseline mortality, we applied regionspecific mortality rates for nonexternal causes (ICD-10 code: A00-R99) to SA-specific population estimates for each age group, thus avoiding the use of highly imprecise SA-specific rate estimates. The regions corresponded to the nine administrative regions of England plus Scotland and Wales, with the mortality rates for 2013 retrieved from NOMIS(ONS (UK Office for National Statistics), 2011b) and the National Records of Scotland (NRS). (NRS (National Records of Scotland), 2024).

### 2.2 Air pollution exposure dataset

The levels of PM_2.5_ and NO_2_ across Great Britain were assigned via a dataset generated by a multi-stage spatio-temporal model (De La Cruz Libardi et al., 2024). Daily concentrations were estimated using a random forest algorithm that combines various local, national and global satellite observations with climate/atmospheric simulations, chemical transport models, land cover features, and road characteristics. The model was trained using observations from ground air quality monitors and then used to predict daily concentrations for each of the 242,851 cells that form a 1 × 1 km grid covering all of Great Britain. The data were merged with the SAs via area-weighted interpolation and aggregated in annual averages from 2008 to 2018. This satellite-based machine learning approach demonstrated good performance, with 10-fold cross-validated R^2^ values of 0.64 overall and 0.66 for the annual averages of PM_2.5_ and NO_2_, respectively.

### 2.3 Computation of premature deaths

We quantified the number of premature deaths attributed to long-term PM_2.5_ and NO_2_ exposure each year by linking the annual concentrations with baseline mortality for each age group in each SA, using relative risk (RR) estimates from published meta-analyses. Specifically, we selected risk estimates of 1.08 (95 % CI: 1.06–1.09) and 1.023 (95 % CI: 1.008–1.037) for a 10 μg/m^3^ increase in PM_2.5_ and NO_2_, respectively. (Chen and Hoek, 2020, COMEAP (Committee on the Medical Effects of Air Pollutants)) For each pollutant *j*, the number of premature deaths *d_iay_* for exposures above a limit value *c* was estimated separately for each SA *i*, age group *a*, and year *y*, via Eq. (1):

(1) $$d_{iayc}=p_{ia}\times m_{ia}\times \left(1-e^{{\hat{\beta }}_{j}{\left(x_{iy}-c\right)}_{+}}\right)$$

The quantities *p_ia_* and *m_ia_* are the population and baseline mortality rates for nonexternal causes, respectively, which are assumed to be stable throughout the study period to ensure comparability across years. The coefficient ${\hat{\beta }}_{j}$ represents the estimated log-RR associated with a unit increase in each pollutant, retrieved from the meta-analyses. (COMEAP (Committee on the Medical Effects of Air Pollutants), Chen and Hoek, 2020) The term *x_iy_* (common to all age groups) represents the overall SA-specific annual air pollutant concentration. The term ${\left(x_{iy}-c\right)}_{+}$ represents the exceedance in the annual exposure above a limit value *c*. This limit value was set to 0 to compute the overall mortality burden, and then increased to compute the premature deaths for different AQ limits. To quantify the burden associated with a range of exceedances, we used values of 5 μg/m^3^, 10 μg/m^3^, 15 μg/m^3^, and 20 μg/m^3^ for PM_2.5_ and 10 μg/m^3^, 20 μg/m^3^, 30 μg/m^3^, and 40 μg/m^3^ for NO_2_.

The age-specific premature deaths and population estimates were combined into standardised excess rates, with the standard European population for 2013 used as a reference. (ONS (UK Office for National Statistics), 2013) The computation was repeated separately for each year and geographical aggregation. The latter were represented by Great Britain as a whole and each of its countries. Empirical confidence intervals (eCIs) of the estimates were obtained via parametric Monte-Carlo simulations. Specifically, a total of 1000 values were sampled from a Gaussian distribution with mean $\hat{\beta }$ and standard deviation derived from the confidence intervals reported in the literature. For each simulated coefficient, we computed the mortality burden at each level of aggregation. All the analyses were performed using the R software (version 4.2.0). (R-Project, 2025).

## 3 Results

**Fig. 1:**
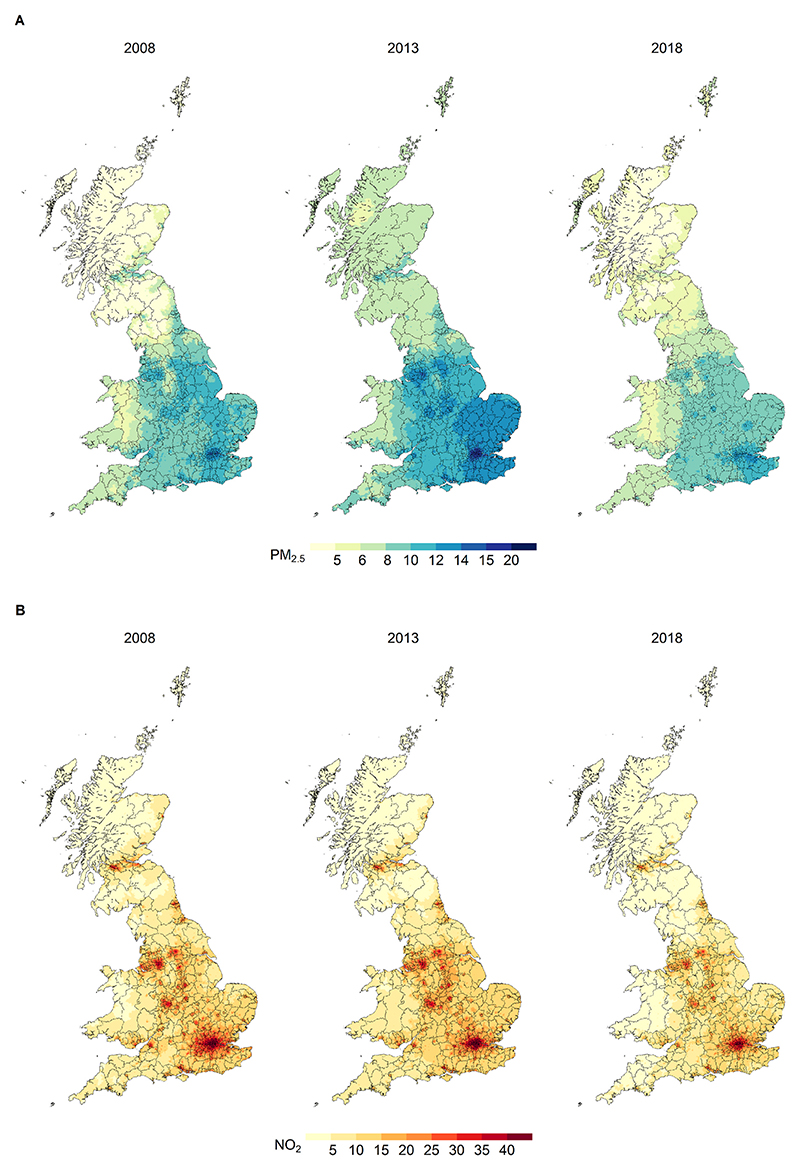
Geographical distribution of annual PM_2.5_ (panel A) and NO_2_ (panel B) across SAs in Great Britain in 2008, 2013, and 2018.

The spatial distributions of PM_2.5_ and NO_2_ levels across Great Britain’s SAs are displayed in Fig. 1 for 2008, 2013 and 2018. The maps reveal strong geographical differences, with high concentrations of both pollutants mostly limited to areas in England. In England, there was a general decreasing trend from 2008 to 2018, although hotspots with relatively high exposure levels remained in urban areas, especially Greater London.

**Fig. 2:**
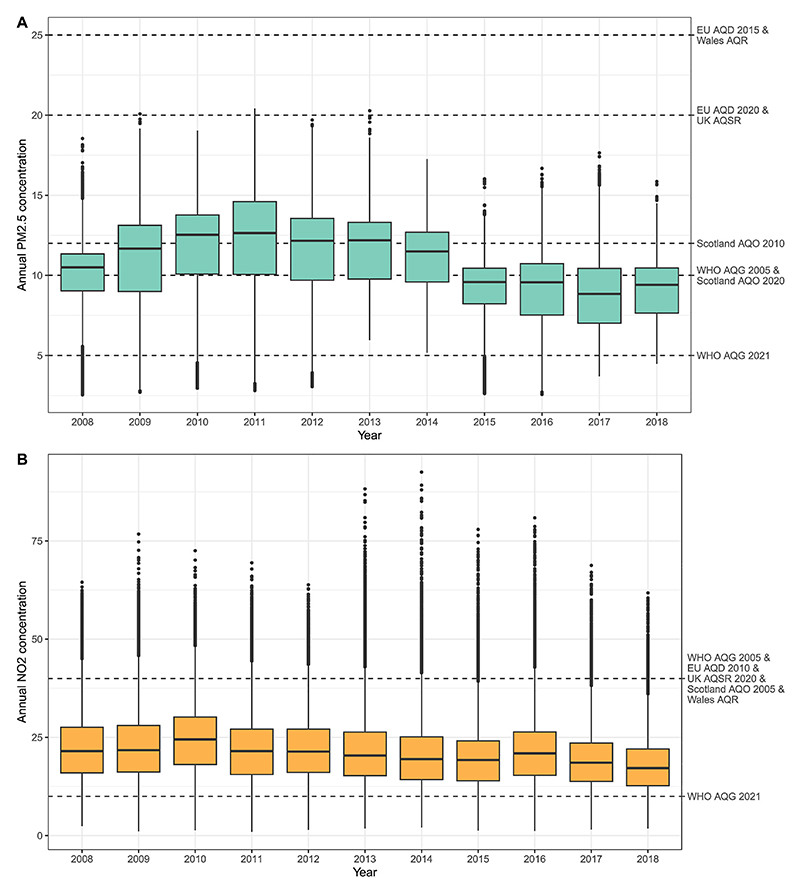
Boxplot of annual PM_2.5_ (panel A) and NO_2_ (panel B) levels from 2008 to 2018. Boxplots contain 41,729 small areas, with annual population-weighted exposures. The labels on the right mark the limits defined in the WHO guidelines and the EU and UK regulations.

Fig. 2 shows the distribution of annual average pollutant exposure across SAs in each year, stratified by country. A decreasing trend in PM_2.5_ is evident across Great Britain, with a noticeable decline after 2014. The annual average NO_2_ exposure was more stable over the study period, with only a slight reduction.

We selected the annual average PM_2.5_ and NO_2_ concentrations in 2008 and 2018 to illustrate the changes in exposure across Great Britain over the study period. Table 1 shows the population-weighted average exposure and the percentages of the population exposed to different concentrations, stratified by country, and rural-urban classification. Across Great Britain, the population-weighted average exposure decreased from 10.5 μg/m^3^ to 9.4 μg/m^3^ and from 23.1 μg/m^3^ to 18.7 μg/m^3^, for PM_2.5_ and NO_2_ respectively. We see an increase in the proportion of the population exposed to the lower concentrations of PM_2.5_ and NO_2_, and a reduction in the proportion exposed to the higher concentration levels. Over the same years, the proportions of the population living in areas exceeding the 2005 WHO thresholds of 10 μg/m^3^ of PM_2.5_ and 40 μg/m^3^ of NO_2_ decreased from 67.6 % to 38.2 % and from 6.3 % to 1.7 %, respectively. These reductions were, however, unevenly distributed across areas and population subgroups. In 2018, the percentage of the population living in areas below the 10 μg/m^3^ PM_2.5_ limit reached 55.8 % in England, whereas it reached 99.4 % in Wales and 100 % in Scotland. For NO_2_, the percentage of the population living in areas below the 40 μg/m^3^ limit in 2018 reached 98.1 % in England, whereas it reached 100 % in Scotland and Wales. In the same year, only 55.3 % of urban areas were below the PM_2.5_ WHO limit and 98 % were below the NO_2_ WHO limit, whereas 91.5 % and 100 % of rural SAs were below the PM_2.5_ WHO limit.

Notably, in 2018, none of the SAs in England and Wales were compliant with the new 2021 WHO AQG of 5 μg/m^3^ for PM_2.5_, and only 10.60 % were compliant with the new NO_2_ level of 10 μg/m^3^. This percentage was only slightly higher in Scotland, with 3.58 % of the SAs below the new PM_2.5_ threshold and 34.55 % below the new NO_2_ threshold.

The annual average PM_2.5_ and NO_2_ concentrations were used to calculate the impact on long-term mortality and then compared across time and geographical areas. Table 2 provides the estimated annual number of premature deaths in 2008 and 2018, stratified by country. In 2008, there were an estimated 40,179 (95 % empirical confidence interval (eCI): 33,599–47,032) premature deaths from PM_2.5_ exposure, while NO_2_ contributed an estimated 24,939 (8836–39,782), across Great Britain. These figures decreased considerably in 2018, with an estimated 36,403 (30,422–42,640) and 20,175 (7125–32,281) premature deaths from PM_2.5_ and NO_2_, respectively.

Table 2 also reports the excess mortality from exposures beyond specific limits. For example, across Great Britain, the quota of premature deaths from exposures beyond the 2005 WHO limits were 3111 (2587–3663) and 206 (72–332) in 2008, and 1360 (1131–1602) and 46 (16–75) in 2018, for PM_2.5_ and NO_2_ respectively.

Table 2 also suggests substantial mortality benefits associated with reducing these limits. For example, limiting the PM_2.5_ concentration to the new WHO AQG of 5 μg/m^3^ could have achieved a much greater benefit of preventing 16,802 (14,000–19,742) premature deaths in 2018. Similarly, for NO_2_, a reduction to the new WHO AQG of 10 μg/m^3^ could have prevented an estimated 8,900 (3,128–14,305) premature deaths in 2018. However, these lower levels are rarely achieved across Great Britain, and compliance with these limits would require substantial effort.

## 4 Discussion

This study provides a comprehensive assessment of premature mortality associated with a range of PM_2.5_ and NO_2_ concentrations across Great Britain from 2008 to 2018. To our knowledge, this is the first study to evaluate health impacts in relation to multiple national and international regulations and guidelines across Great Britain, and to report detailed country-wide results at different aggregations.

The results indicate a strong reduction in the concentration of both pollutants across Great Britain. In 2018, PM_2.5_ levels were below the UK AQS limit of 20 μg/m^3^ for all 41,729 SAs, although a substantial proportion of people living in England and Wales were still exposed to concentrations higher than 10 μg/m^3^, a recommended limit set by the previous WHO AQGs in 2005. The NO_2_ concentration exceeded the UK AQS limit of 40 μg/m^3^ for 615 SAs in 2018. Higher levels of both pollutants occurred primarily in England and in metropolitan areas.

Across Great Britain, exposure to PM_2.5_ and NO_2_ contributed 36,403 and 20,175 premature deaths, respectively, in 2018. The results of this study suggest that modest reductions in the PM_2.5_ concentration to 15 or 10 μg/m^3^ would have limited mortality benefit, whereas more ambitious directives to cut concentrations beyond the 5 μg/m^3^ value now recommended by the WHO, could reduce this toll by 46 %. Similarly, a reduction in the NO_2_ concentration limit to 30 or 20 μg/m^3^ would provide some benefit, but adherence to the recommended limit of 10 μg/m^3^ could reduce premature deaths by 44 %.

However, in 2018, no area in England or Wales was compliant with the 2021 WHO AQG of 5 μg/m^3^ for PM_2.5_, and only 10.6 % were compliant with the 10 μg/m^3^ level for NO_2_. Therefore, implementing the WHO recommendations as new legal limits would require bold political decisions and substantial effort. The 2025 report on emissions of air pollutants in the UK indicates that major contributions of PM_2.5_ and nitrogen oxides came from a range of sectors. (DEFRA (UK Department for Environment Food & Rural Affairs), 2025c, DEFRA (UK Department for Environment Food & Rural Affairs), 2025b, DEFRA (UK Department for Environment Food & Rural Affairs), 2025a) Domestic combustion, such as household wood and coal burning, contributed 20 % of total PM_2.5_ emissions, while road transport contributed 21 %. (DEFRA (UK Department for Environment Food & Rural Affairs), 2025c) Road transport contributed a greater proportion of NO_2_ emissions (30 %); while the contribution from energy industries was also high (19 %). (DEFRA (UK Department for Environment Food & Rural Affairs), 2025b) Although these findings suggest that coordinated action across several sectors is required to achieve substantial reductions, there are potential multipollutant benefits to targeted policies within each sector.

Additional challenges include air pollution in the form of transboundary contamination, as well as natural sources, both of which contribute to overall concentrations and are difficult to remove. Therefore, a reduction of the scale required to meet the new WHO guidelines will likely demand consistent plans to evaluate the contributions and important cuts to emissions from multiple sectors.

The findings from this study are consistent but not identical to those reported in the literature. The UKHSA Air Quality and Public Health Group estimated a burden of between 29,000 and 43,000 deaths for adults aged 30 years and over due to long-term PM_2.5_ and NO_2_ exposure in the UK in 2019, (UKHSA (UK Health Security Agency), 2022) with between 26,000 and 38,000 deaths in England. The UK’s Committee on the Medical Effects of Air Pollutants (COMEAP) reported that the 2008 anthropogenic PM_2.5_ was responsible for nearly 29,000 deaths in those above 30 years of age in the UK. (UK Government, 2018) Milojevic and colleagues quantified the years of life lost attributed to long-term PM_2.5_ exposure by socioeconomic deprivation and urban-rural status across England in 2010, indicating a greater burden in urban areas and, to a lesser extent, in the most deprived groups (Milojevic et al., 2017). While Macintyre and colleagues predicted that the proportion of the UK population exposed to PM_2.5_ and NO_2_ would decline in future, an estimated 26,287 to 42,442 deaths were attributable to PM_2.5_ and NO_2_ in 2018, with those living in London, the north west and south east carrying the greatest mortality burden (Macintyre et al., 2023). Finally, Carnell and colleagues assessed trends from the 1970s–2010s across the UK, detecting reductions in both pollutants following the introduction of the first National AQ Strategy in 1997 and decreases in the attributable fraction of deaths from 11.83 % to 5.21 % (for PM_2.5_) and from 5.32 % to 2.96 % (for NO_2_) (Carnell et al., 2019). However, none of these studies evaluated the burden in relation to specific concentration limits.

Other investigations focused on specific geographical areas; for example, Khomenko and colleagues reported 107 (95 % CI: 70–144) PM_2.5_-related and 5 (0–13) NO_2_-related excess deaths in London in 2015 for exposure levels higher than the 2005 WHO guidelines (Khomenko et al., 2021). Dajnak and colleagues reported an overall combined effect for PM_2.5_ and NO_2_ equivalent to between 3598 and 4096 deaths in people aged 30 years and older in 2019 in London (Dajnak et al., 2021).

The different results across these studies can be attributed to various factors, including different analytical methods, population groups, periods/years, and concentration-response coefficients from the literature. However, the most important factor is the derivation of the exposure concentration levels used to quantify the health burden, which can vary in resolution and accuracy.

Our assessment has several advantages. The study assigned pollutant concentrations and computed premature mortality for each of the 41,729 SAs in Great Britain, thus offering a comprehensive picture of the exposure levels and health burdens across the populations of England, Wales, and Scotland. This detailed analysis was made possible by using high-resolution PM_2.5_ and NO_2_ maps reconstructed over a 1 × 1 km grid via a spatiotemporal machine learning model characterised by relatively high accuracy. Estimates of the mortality burden were stratified by age groups and reported both as absolute numbers of deaths and standardised rates, therefore accounting for demographic differences across areas. The analysis covers a period of 11 years and evaluates the exposure levels and related health impacts in relation to current air quality limits and potential revisions.

However, some limitations must be acknowledged. First, the exposure levels are based on modelled data and not direct measurements, and while the spatiotemporal model shows good performance, it is possible that pollutant concentrations are inaccurately reconstructed in some areas or periods. The standardised rates and premature deaths should be interpreted with caution since they were estimated using ambient concentration levels and not personal exposure, although this is consistent with the estimated risk in most studies included in the meta-analyses. Second, the mortality rates and populations were kept constant to facilitate a consistent comparison across years, a choice that can lead to small biases in estimated excess mortality at each end of the study period. While this a recognised limitation of this study, it should be noted that figures published by the UK Office for National Statistics suggest a minimal change in overall mortality rate over this period. (ONS (UK Office for National Statistics), 2025) The health impact assessment was performed via several assumptions, including the use of distinct estimated exposure-response relationships between long-term exposure and mortality for each pollutant from recent meta-analyses. (Chen and Hoek, 2020, COMEAP (Committee on the Medical Effects of Air Pollutants)) This step assumes that this level of risk, obtained from internationally pooled data, applies identically across all strata of the UK population, and does not account for potential heterogeneity in the associated health risks across SAs and across age groups. In using these effect estimates, we also assume that they reflect the risk profile of the population throughout the study period, regardless of any population-level changes in risk. Relatedly, the calculation is based on regional mortality rates, without considering differences in baseline mortality. There are also differences in the uncertainty of the estimated mortality burden; the NO_2_ estimates having lower accuracy than those for PM_2.5_. This outlines the need for further epidemiological evidence on the risks of long-term exposure to NO_2_. Finally, a limitation common to air pollution studies should be noted; concentrations of pollutants are often highly correlated, and it is therefore difficult to unequivocally attribute health effects to each individual pollutant. The calculation of a combined mortality burden across pollutants should therefore consider this correlation.

## 5 Conclusions

This study offers a comprehensive assessment of the outdoor concentrations of PM_2.5_ and NO_2_ and related premature mortality across Great Britain, indicating that while improvements in air quality have been obtained in the last decade, exposure to these pollutants is still responsible for a substantial health burden. Although adherence to the most recent WHO guidelines would require ambitious policy strategies, particularly in urban areas, our analysis suggests that reducing the current legal limits would result in important health benefits.

## List of abbreviations

Abbreviation: Definition
AQ: Air quality
AQD: Air Quality Directive
AQGs: Air Quality Guidelines
AQS: Air Quality Standards
COMEAP: Committee on the Medical Effects of Air Pollutants
DZ: Data Zone
EU: European Union
LSOA: Lower layer Super Output Area
NO_2_: Nitrogen dioxide
NRS: National Records of Scotland
PM_2.5_: Fine particulate matter with an aerodynamic diameter smaller than 2.5 μm
SA: Small area
UK: United Kingdom
UKHSA: UK Health Security Agency
WHO: World Health Organization

## Supplementary Material

Supplementary data to this article can be found online at https://doi.org/10.1016/j.apr.2025.102827.

Appendix

## Figures and Tables

**Table 1:** Total population, mean pollutant concentration, and percentage exposed to different PM_2.5_ (≤5, 5–10, 10–15, 15–20,≥20) and NO_2_ (≤10, 10–20, 20–30, 30–40,≥40) level ranges in Great Britain for 2008 and 2018, by country.

Spatial aggregation	Total population	Year	Mean PM_2.5_ (μg/ m^3^)	% Population exposed to PM_2.5_ (μg/m^3^)					Mean NO_2_ (μg/ m^3^)	% Population exposed to NO_2_ (μg/m^3^)				
				PM_2.5_ ≤ 5	5 < PM_2.5_ ≤ 10	10 < PM_2.5_≤ 15	15 < PM_2.5_ ≤ 20	PM_2.5_ > 20		NO_2_ ≤ 10	10 < NO_2_ ≤ 20	20 < NO_2_ ≤ 30	30 < NO_2_ ≤ 40	NO_2_ > 40
Great	61,371,315	2008	10.5	1.60 %	30.80 %	67.20 %	0.40 %	0.00 %	23.1	6.50 %	33.70 %	39.40 %	14.10 %	6.30 %
Britain		2018	9.4	0.30 %	61.50 %	38.20 %	0.00 %	0.00 %	18.7	12.70 %	50.60 %	26.50 %	8.50 %	1.70 %
England	53,012,456	2008	10.9	0.20 %	23.10 %	76.20 %	0.50 %	0.00 %	24.1	4.40 %	31.30 %	41.30 %	15.70 %	7.30 %
		2018	9.9	0.00 %	55.80 %	44.20 %	0.00 %	0.00 %	19.6	9.00 %	50.50 %	28.70 %	9.80 %	1.90 %
Scotland	5,295,403	2008	7.3	16.70 %	82.00 %	1.30 %	0.00 %	0.00 %	17.7	17.20 %	44.20 %	32.50 %	5.90 %	0.30 %
		2018	6.2	3.60 %	96.40 %	0.00 %	0.00 %	0.00 %	13.4	33.90 %	49.90 %	16.00 %	0.10 %	0.00 %
Wales	3,063,456	2008	8.8	0.00 %	75.80 %	24.20 %	0.00 %	0.00 %	14.8	24.60 %	55.40 %	19.20 %	0.80 %	0.00 %
		2018	7.6	0.00 %	99.40 %	0.60 %	0.00 %	0.00 %	11.8	40.20 %	53.00 %	6.80 %	0.00 %	0.00 %

**Table 2:** Estimated premature deaths due to the average annual concentrations exceeding certain concentration limits (5, 10, 15, and 20 μg/m^3^ for PM_2.5_ and 10, 20, 30 and 40 μg/m^3^ for NO_2_), in 2008 and 2018.

Location	Total Population	Year	Excess mortality to PM_2.5_ (in μg/m^3^)					Excess mortality to NO_2_ (in μg/m^3^)				
			Overall PM_2.5_	PM_2.5_ > 5	PM_2.5_ > 10	PM_2.5_ > 15	PM_2.5_ > 20	Overall NO_2_	NO_2_ > 10	NO_2_ > 20	NO_2_ > 30	NO_2_ > 40
Great Britain	61,371,315	2008	40,179 (33,599−47,032)	20,810 (17,350−24,437)	3111 (2587−3663)	6 (5−7)	0 (0−0)	24,939 (88,36−39,782)	13,446 (4739−21,556)	4648 (1633−7472)	1079 (379−1736)	206(72−332)
		2018	36,403 (30,422−42,640)	16,802 (14,000−19,742)	1360 (1131−1602)	0 (0−0)	0 (0−0)	20,175 (7125−32,281)	8900 (3128−14,305)	2042 (717−3287)	393 (138−633)	46 (16−75)
England	53,012,456	2008	35,308 (29,531−41,321)	19,015 (15,855−22,326)	3065 (2549−3608)	6 (5−7)	0 (0−0)	21,924 (7772−34,954)	12,186 (4297−19,529)	4387 (1542−7051)	1061 (372−1707)	206 (72−331)
		2018	32,166 (26,887−37,668)	15,745 (13,121−18,498)	1360 (1131−1602)	0 (0−0)	0 (0−0)	17,845 (6305−28,540)	8191 (2880−13,162)	1990 (698−3203)	393 (138−633)	46 (16−75)
Scotland	5,295,403	2008 2018	2905 (2425−3407) 2512 (2095−2949)	959 (798−1128) 472 (392−555)	2 (1−2) 0 (0−0)	0 (0−0) 0 (0−0)	0 (0−0) 0 (0−0)	2054 (725−3287) 1564 (551−2510)	923 (324−1484) 517 (181−833)	224 (78−361) 47 (16−76)	18 (6−29) 0 (0−0)	0 (0−1) 0 (0−0)
Wales	3,063,456	2008 2018	1966(1642−2304) 1724 (1439−2022)	837 (697−983) 585 (487−689)	45 (37−53) 0 (0−0)	0 (0−0) 0 (0−0)	0 (0−0) 0 (0−0)	961 (338−1542) 766 (269−1231)	337 (118−543) 192 (67−310)	37 (13−60) 5 (2−9)	0 (0−0) 0 (0−0)	0 (0−0) 0 (0−0)

## Data Availability

The population estimates for each small area (i.e., the LSOA in England and Wales, and data zones in Scotland) were obtained from NOMIS (www.nomisweb.co.uk) and the National Records of Scotland (www.nrscotland.gov.uk). Information on the small-area characteristics (e.g., urban-rural classification) was gathered from the following public sources: www.gov.uk and www.gov.scot. Region-specific mortality rates for nonexternal causes (ICD-10 code: A00-R99) were retrieved from NOMIS and the National Records of Scotland. Annual air pollution exposures over the 1 × 1 km grid were gathered from a multistage spatiotemporal model developed by De La Cruz Libardi et al. (De La Cruz Libardi et al., 2024)
