# Supplementary material for: Mortality impacts of long-term PM2.5 and NO2 exposure in Great Britain under national and international air quality limits
Source: Atmos Pollut Res. Author manuscript; Available in PMC 2026 Apr 8. (PMC7618988; doi:10.1016/j.apr.2025.102827)
Supplement: Appendix [file EMS213206-supplement-Appendix.pdf]

Supplementary Material

Mortality impacts of long-term $PM_{2.5}$ and NO_2 exposure in Great Britain under national and international air quality limits

Gillian Flower*[1,2], Rochelle Schneider*[3,4], Karen Exley[5], Christina Mitsakou[5], Pierre Masselot[1], and Antonio Gasparrini[1]

[1] Environment & Health Modelling (EHM) Lab, Department of Public Health, Environments and Society, London School of Hygiene & Tropical Medicine, London, United Kingdom

[2] Department for Health and Social Care, UK government

[3] Φ-lab, European Space Agency (ESA), Frascati, Italy

[4] Epidemiology and Population Health, London School of Hygiene & Tropical Medicine, London, United Kingdom

[5] Air Quality and Public Health, Environmental Hazards and Emergencies department, UK Health Security Agency

National and International Air Quality Limits

$PM_{2.5}$ and NO_2 annual average concentration limits from the following national and international organisations:

- The World Health Organization (WHO) Air Quality Guidelines (AQG)
- The European Union (EU) Air Quality Directive (AQD)
- The United Kingdom (UK) Air Quality Standards Regulations (AQSR) and Environmental Targets (Fine Particulate Matter) Regulations (ETR)
- The Air Quality Scotland Regulations (AQSR)
- The Air Quality Wales Regulations (AQWR)

Table S1 | Annual average air pollution concentration guidelines and regulations applicable to $PM_{2.5}$ and NO_2

**Pollutant**	**Directive**	**Date**	**Annual Average Concentration** (μg/m^3)
$PM_{2.5}$	WHO AQG (WHO (World Health Organization), 2015, WHO (World Health Organization), 2005)	2021	5
		2005	10
	EU AQD (EU (European Union), 2008)	2020	20
		2015	25
	UK AQSR (DEFRA (UK Department for Environment Food & Rural Affairs), 2010, DEFRA (UK Department for Environment Food & Rural Affairs), 2023)	2020	20[a]
	UK ETR (UK Government, 2023)	2040	10
	Scotland AQSR (Scotland's Environment, 2021)	2020	10
	Wales AQWR (Welsh Government, 2002)	2000	25
NO_2	WHO AQG (WHO (World Health Organization), 2015, WHO (World Health Organization), 2005)	2021	10
		2005	40
	EU AQD (EU (European Union), 2008)	2010	40
	UK AQSR (DEFRA (UK Department for Environment Food & Rural Affairs), 2010, DEFRA (UK Department for Environment Food & Rural Affairs), 2023)	2005	40
	Scotland AQSR (Scotland's Environment, 2021)	2005	40
	Wales AQWR (Welsh Government, 2002)	2000	40

Additionally, the UK ETR set a population exposure reduction target of 35% for $PM_{2.5}$, to be met by 2040.(UK Government, 2023)

[a] Applicable to England, Wales and Northern Ireland

References

DEFRA (UK Department for Environment Food & Rural Affairs). 2010. *UK Air Quality Limits* [Online]. Available: https://uk-air.defra.gov.uk/air-pollution/uk-limits [Accessed].

DEFRA (UK Department for Environment Food & Rural Affairs). 2023. *England Fine Particulate Matter Targets* [Online]. Available: https://uk-air.defra.gov.uk/pm25targets/overview/index [Accessed].

EU (European Union). 2008. *Directive 2008/50/EC of the European Parliament and of the Council of 21 May 2008 on ambient air quality and cleaner air for Europe. 152 vol. OJ L (2008)* [Online]. Available: https://eur-lex.europa.eu/eli/dir/2008/50/oj/eng [Accessed].

Scotland's Environment. 2021. *Air Quality in Scotland - Air Quality Standards and Objectives* [Online]. Available: https://www.scottishairquality.scot/air-quality/standards [Accessed].

UK Government. 2023. *The Environmental Targets (Fine Particulate Matter) (England) Regulations 2023* [Online]. Available: https://www.legislation.gov.uk/uksi/2023/96/contents/made [Accessed].

Welsh Government. 2002. *Air Quality in Wales - Standards and Objectives* [Online]. Available: https://www.airquality.gov.wales/about-air-quality/standards-and-objectives [Accessed].

WHO (World Health Organization). 2005. *Air quality guidelines global update 2005* [Online]. World Health Organization. Available: https://www.who.int/publications/i/item/WHO-SDE-PHE-OEH-06.02 [Accessed].

WHO (World Health Organization). 2015. *WHO expert consultation: available evidence for the future update of the WHO Global Air Quality Guidelines (AQGs)* [Online]. Available: https://www.who.int/europe/publications/i/item/WHO-EURO-2016-4105-43864-61762 [Accessed].
